# Long-Term Multimodal Imaging Analysis of Selective Retina Therapy Laser Lesions

**DOI:** 10.3390/life13040886

**Published:** 2023-03-27

**Authors:** Maximilian Binter, Migle Lindziute, Christopher Rosenstein, Carsten Framme, Jan Tode

**Affiliations:** Department of Ophthalmology, Hannover Medical School, 30625 Hannover, Germany; lindziute.migle@mh-hannover.de (M.L.); rosenstein.christopher@mh-hannover.de (C.R.); framme.carsten@mh-hannover.de (C.F.); tode.jan@mh-hannover.de (J.T.)

**Keywords:** selective retina treatment, central serous chorioretinopathy, micropulse laser, subretinal fluid, fluorescein angiography, OCT, fundus autofluorescence

## Abstract

This study evaluates the long-term effects of selective retina therapy (SRT) on the retinal pigment epithelium (RPE) and neuroretina in patients with central serous chorioretinopathy. SRT was performed on 36 patients using a Nd:YLF-Laser at 527 nm (R:GEN^®^, Lutronic, Goyang-Si, Republic of Korea). A total of 994 titration spots were examined using up to three years’ multimodal imaging. Leakage in fluorescein angiography (FA) was observed after SRT in 523 lesions and resolved after one month. SRT lesions were not visible clinically, but appeared as brightly reflective areas in infrared and multicolor images. Normal morphology was observed in optical coherence tomography (OCT) immediately after SRT. After one month, thickening of the RPE and interdigitation zone changes were seen and disappeared after 539 ± 308 days. No RPE atrophies occurred during the observation period. Decreased fundus autofluorescence (FAF) was mostly observed directly after SRT followed by increased FAF at one month, which faded over time. A significant decrease in the number of visible lesions in the FA and FAF was observed within the three-year follow-up. OCT findings are consistent with animal studies showing SRT-related defect closure by hypertrophy and migration of neighboring cells without RPE atrophy or photoreceptor damage. This suggests that SRT is a safe treatment option for macular diseases and does not lead to retinal atrophy.

## 1. Introduction

Conventional retinal photocoagulation (PC) is an established therapy that has become part of the standard treatment for various retinal and choroidal diseases, especially in diabetic retinopathy [1]. The energy is converted into heat, leading to thermal damage of the retinal pigment epithelium (RPE), neuroretina and the choroid [2]. A side effect of this therapy is irreversible damage to the retinal structures and associated scotomas as well as the development of RPE-related atrophic areas [3]. This is particularly problematic in the treatment of macular pathologies, especially because the developing atrophic areas can expand over time and localized parafoveal laser foci can involve the fovea leading to a central scotoma [4].

To avoid these undesirable side effects of laser therapy, photodisruptive selective retina therapy (SRT) with sparing of the photoreceptor layer was developed. SRT uses pulse durations below 5 µs, which results in selective RPE cell disruption by intracellular microbubble formation and does not affect the neuroretina or choroid. The microvaporization-induced local RPE defects are closed by the proliferation and migration of neighboring pigment epithelial cells [5]. It is postulated that these remodeling processes contribute to the restructuring of the blood–retinal barrier, the improvement of metabolic functions of the RPE and therefore act as a rejuvenating therapy for the RPE [6]. The regenerating RPE cells subsequently form a functional closed tissue structure. This defect closure in the treated area occurs usually within one to two weeks after irradiation [7,8,9]. SRT has shown therapeutic success in central serous chorioretinopathy and diabetic macular edema [8,10,11,12]. No side effects or complications of this procedure have been reported so far [5,6,12,13]. One problem in the clinical application of SRT is that neither the optimal irradiation parameters nor the standard treatment strategies regarding the application geometry of laser foci, the number of spots and repetitive treatment regimens have been reported and so far are only based on common therapeutic regimes of conventional laser coagulation treatment [8]. To facilitate the procedure, an SRT system with real-time feedback technology was developed with the idea of auto-stopping the laser when the microbubble formation is detected [7,14].

Central serous chorioretinopathy (CSC) is a common disease of the posterior segment of the eye, which typically presents with a dome-shaped neuroretinal detachment due to accumulation of subretinal fluid (SRF), retinal pigment epithelium (RPE) dysfunction, and hyperpermeability and thickening of the underlying choroid. Patients with CSC suffer from central vision loss, scotomas, micropsia or metamorphopsia, hypermetropisation, and reduced contrast sensitivity [15]. The complex clinical picture and not conclusively clarified pathogenesis of this disease complicate the search for an optimal treatment method. To avoid long-term RPE damage caused by SRF, complete resolution of all fluid should be the primary goal of treatment; however, due to the high spontaneous remission rate with good visual prognosis, therapy should demonstrate a good safety profile [16]. Peripheral retinal pathologies that present with leakage are commonly treated using thermal laser photocoagulation. However, due to the associated visual field defects caused by the destruction of the neurosensory retina, macular treatment by conventional thermal laser photocoagulation is problematic in CSC and contraindicated in the fovea [10]. Half-dose photodynamic therapy (PDT), involving intravenous administration of verteporfin (Visudyne^®^ by Novartis, Basel, Switzerland), has proven to be a successful treatment for CSC [17]. However, due to the persistent global verteporfin shortage, which remains a long-term concern, alternative treatments are urgently needed [18]. SRT has already demonstrated efficacy in clinical studies, including its use in treating foveal central serous chorioretinopathy cases [19,20]. Furthermore, a network meta-analysis indirectly compared SRT with a placebo for CSC, and found a significant effect on the resolution of subretinal fluid [21].

Some studies investigating the safety aspects of SRT already found no adverse effects during the observation period. Studies in which electrophysiological examinations were performed after SRT found no changes in electroretinography (ERG) and multifocal ERG, suggesting the absence of photoreceptor damage [2,22]. Furthermore, microperimetry after SRT showed no loss of retinal sensitivity and thus no scotomas [20,23]. Additionally, the oxygen consumption of photoreceptors remained unchanged after SRT, indicating that their function and metabolism were unaltered [24].

An important factor regarding safety of the treatment is the long-term effect. In conventional laser therapy, the zone of lethal damage is not necessarily visible initially; however, as the laser-induced RPE atrophy continues to expand for years, a phenomenon called atrophic creep is observable [25,26]. In this study, the long-term effect of SRT on the human retina was evaluated using multimodal imaging over a period of three years.

## 2. Material and Methods

### 2.1. Subject Selection

The local ethics committee of the Hannover Medical School approved this retrospective study (vote number 7393) and the principles of the Declaration of Helsinki were followed in the study’s conduct. Patients with chronic CSC (cCSC), who showed visible macular leakage spots in fluorescein angiography and received SRT, were included in this study. All patients underwent a detailed ophthalmologic examination including determination of best-corrected visual acuity (BCVA), slit-lamp examination, tonometry, and indirect ophthalmoscopy before enrollment in the study. Fluorescein angiography (FA), fundus autofluorescence (FAF), fundus photography, multicolor imaging and optical coherence tomography (OCT) at the baseline and follow-up visits were performed using Heidelberg Engineering Spectralis OCT (Heidelberg Engineering, Heidelberg, Germany). Patients were examined at the baseline, one and six months as well as one, two, and three years after SRT.

### 2.2. SRT Protocol

Treatment was performed using a slit-lamp-based laser (R:GEN^®^, Lutronic, Goyang-Si, Republic of Korea, CE-certified according to 93/42 MDD) that meets the requirements of the European Union Medical Device Regulation 93/42 EEC. It is a frequency-doubled Nd: YLF laser with a wavelength of 527 nm. The treatment was performed using an irradiation duration of 150 ms at 100 Hz frequency with a pulse duration of 1.7 µs and a lesion size of 200 µm. Every SRT exposure consisted of a sequence of 15 pulses. Before performance of the macular treatment, titration lesions were applied along the vascular arches outside the macula in ascending intensity with a starting energy of 50 µJ per lesion increasing in 10 µJ steps until a visible whitening effect was observed funduscopically. This whitening reaction vanished again within a few minutes. For each energy level during titration, two spots were applied. During the administration of each spot, energy was controlled by a ramping mode using optoacoustic feedback. No auto-stop function was used. A 20% lower energy than the one needed to produce a whitening effect was used for the final treatment of the leakage spots. The titration was used to ensure sufficient energy levels for RPE damage. FA was performed directly after treatment with SRT [5,10].

### 2.3. Test Lesion Analysis

Data from follow-up examinations were analyzed. Visible titration lesions in FA and FAF were counted. Procedure sketches were made marking the energy used and the occurrence of a visible whitening reaction. The titration lesions were then compared and matched to the ones shown in the sketches. Horizontal and vertical diameters of lesions, corresponding to the treatment energy and lesions where visual whitening effect for titration purposes was observed, were measured manually. Dense-volume SD-OCT scans of the lesions were performed and the morphology of the RPE and the overlying retina was analyzed. Titration lesions were chosen for analysis to measure the effects of SRT treatment on healthy retina. Macular treatment foci were not included in the analysis because it is not possible to distinguish between the effect of SRT and alterations caused by cCSC. SRT test lesions in retinal areas altered by cCSC were counted but not measured or analyzed in SD-OCT. This mainly concerned titration lesions at the inferior vascular arcade, because this region is more frequently affected due to gravitational phenomena [15].

### 2.4. Statistical Analysis

The area of SRT lesions was calculated approximately as an oval using the formula A = r1 × r2 × π (A = lesion area; r1 = lesion radius one and r2 = lesion radius two). Data obtained in the study were statistically analyzed using Microsoft Excel 2019 (Microsoft Corporation, Redmond, WA, USA) and the Statistical Package for Social Sciences for Windows (SPSS) version 27.0 (IBM Corp., Armonk, NY, USA). Images were created using a GraphPad Prism Ver. 8.4.3 (GraphPad Software Inc., La Jolla, CA, USA).

The one-sample binomial test was used to determine if the proportion of subjects in one of two categories was significantly different from 0.5. The Kolmogorov–Smirnov test was used to analyze the normality of the distribution of empirical data. However, in smaller sample sizes (*n* < 50) the Shapiro–Wilk test was used to examine the distribution’s normality. The Wilcoxon rank test was used to compare the means of matched samples in not normally distributed data. The Mann–Whitney U Test was used to compare the equality of two independent samples with not normally distributed data. The Spearman correlation coefficient was used to measure the linear correlation between variables. All means are presented with standard deviations (Mean ± SD). Not normally distributed data were also presented using the median with interquartile range Median (IQR). Results with a significance level of *p* < 0.05 were interpreted as statistically significant.

## 3. Results

Our study followed up 36 subjects (eyes) of 33 patients with central serous chorioretinopathy who were treated with SRT. All of these eyes were monitored for one year, 20 (56%) for up to two years, and 10 (28%) for up to three years.

### 3.1. Demographic Data

A total of 29 (88%) males and four (12%) females affected with CSC was included in the study. Significantly more males were affected (*p* > 0.001). The right eye was affected in 13 (36%) cases while the left eye was affected in 23 (64%) cases (*p* = 0.132). The average age of subjects was 46.2 ± 8.6 years.

### 3.2. SRT Energy Characteristics

The average energy needed to see a visible whitening reaction for titration purposes was 179.4 ± 29.0 µJ (minimum 110 µJ, maximum 220 µJ). The average energy used for macular treatment was 143.3 ± 21.7 µJ (minimum 90 µJ, maximum 180 µJ).

### 3.3. Morphological Changes

**Figure 1:**
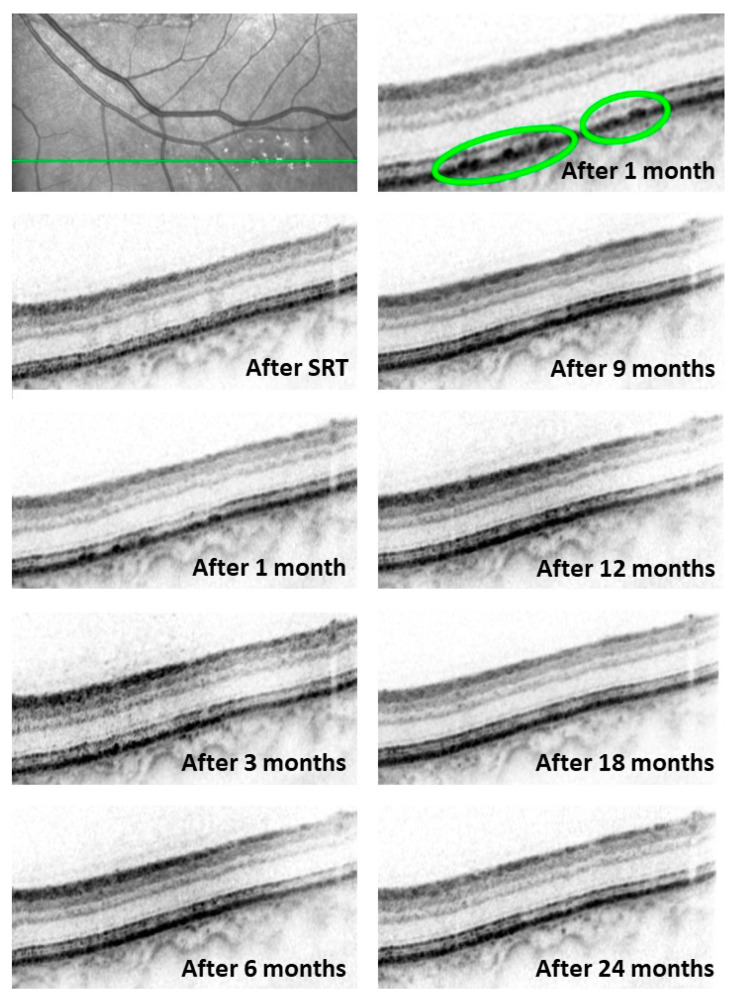
SD-OCT images of the test lesions over 24 months. The top row shows the infrared image of the depicted area (green line) and an enhanced image section of the titration lesions 1 month after SRT. Typical dune-shaped RPE changes are clearly visible in the enhanced image section (circled). Irregularities within the RPE layer and the interdigitation zone are still visible at the 9-month follow-up examination. However, the RPE layer fully reverted to its normal morphology at 18 months.

No changes in SD-OCT scans of the titration lesions were observed immediately after performing the SRT procedures. Dune-shaped thickenings of the RPE and consecutive changes in the interdigitation zone were seen one month after treatment. In some cases, isolated gaps in the RPE layer were seen adjacent to the dune-shaped thickenings. These changes in the RPE layer regressed over time and returned to a normal OCT morphology after 11.5 ± 9.3 months. Changes within the interdigitation zone disappeared after 17.7 ± 10.1 days and therefore could be observed for a longer period than those in the RPE layer (*p* = 0.0033). Changes in the RPE and interdigitation zone morphology at the baseline and over 24 months are presented in Figure 1.

**Figure 2:**
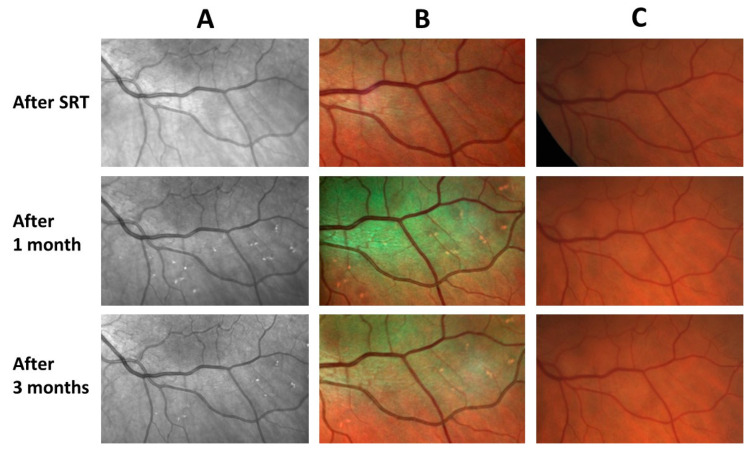
Infrared images (**A**), multicolor images (**B**) and fundus photographs (**C**) of the SRT test lesions at baseline, 1-month and 3-month follow-up examinations. No lesions are visible in any of these recordings directly after performing the SRT. The foci appear in the infrared image (**A**) and multicolor images (**B**) one month after the irradiation and start to fade over time. It is not possible to detect the lesions at any time in fundus photography (**C**).

The SRT lesions were not visible either clinically or in the fundus photography. The lesions appeared as brightly reflecting areas in infrared and multicolor images (Figure 2).

**Figure 3:**
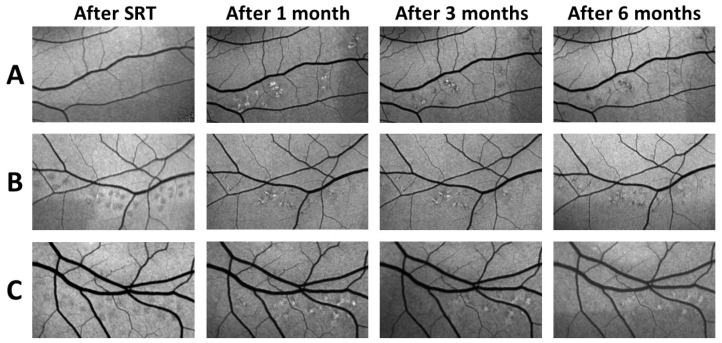
FAF images of SRT test lesions of 3 different patients (**A**–**C**) taken over a period of 6 months. Decreased autofluorescence often appeared in the area of irradiation directly after SRT (**B**,**C**) but this effect is absent in some cases (**A**). Increased autofluorescence is clearly visible 1 month after SRT. This can be very apparent (**C**) or interspersed with more (**B**) or less (**A**) decreased autofluorescent areas. This increase in FAF fades completely (**A**) or only slightly (**B**) over time. However, in some patients it remains constant (**C**).

**Figure 4:**
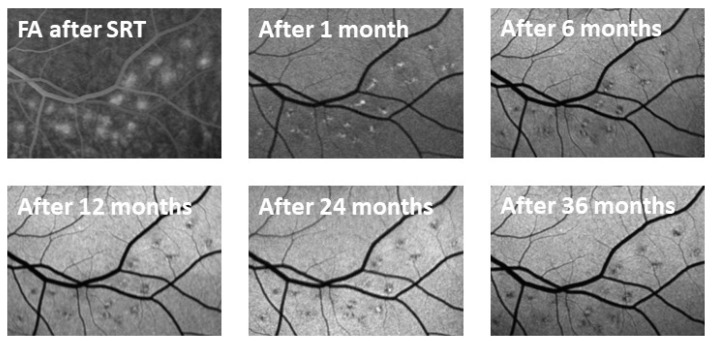
FAF images of SRT test lesions over 3 years. The first image shows leakage of the lesions in FA after treatment. Increased autofluorescence is visible 1 month after treatment. Later, a mainly decreased autofluorescence with interspersed increased autofluorescence is observed. No RPE atrophy was observed over time.

Fundus autofluorescence showed a diverse pattern of alterations induced by SRT which varied from patient to patient. In most subjects, areas of increased autofluorescence were observed immediately after SRT; however, in some patients this effect was absent. One month later, these lesions appeared as areas of increased FAF with partial decreased FAF. In the following examinations, different patterns were observed. In some cases, the foci faded continuously; in some patients, the lesions with mixed increased and decreased FAF faded gradually, whereas in others a constant increase in autofluorescence persisted but subsided over time. However, most patients showed reduced FAF in the later follow-ups (Figure 3). In the further follow-up period, the changes remained constant or regressed. Furthermore, no RPE atrophies developed in the observed period of three years (Figure 4).

**Figure 5:**
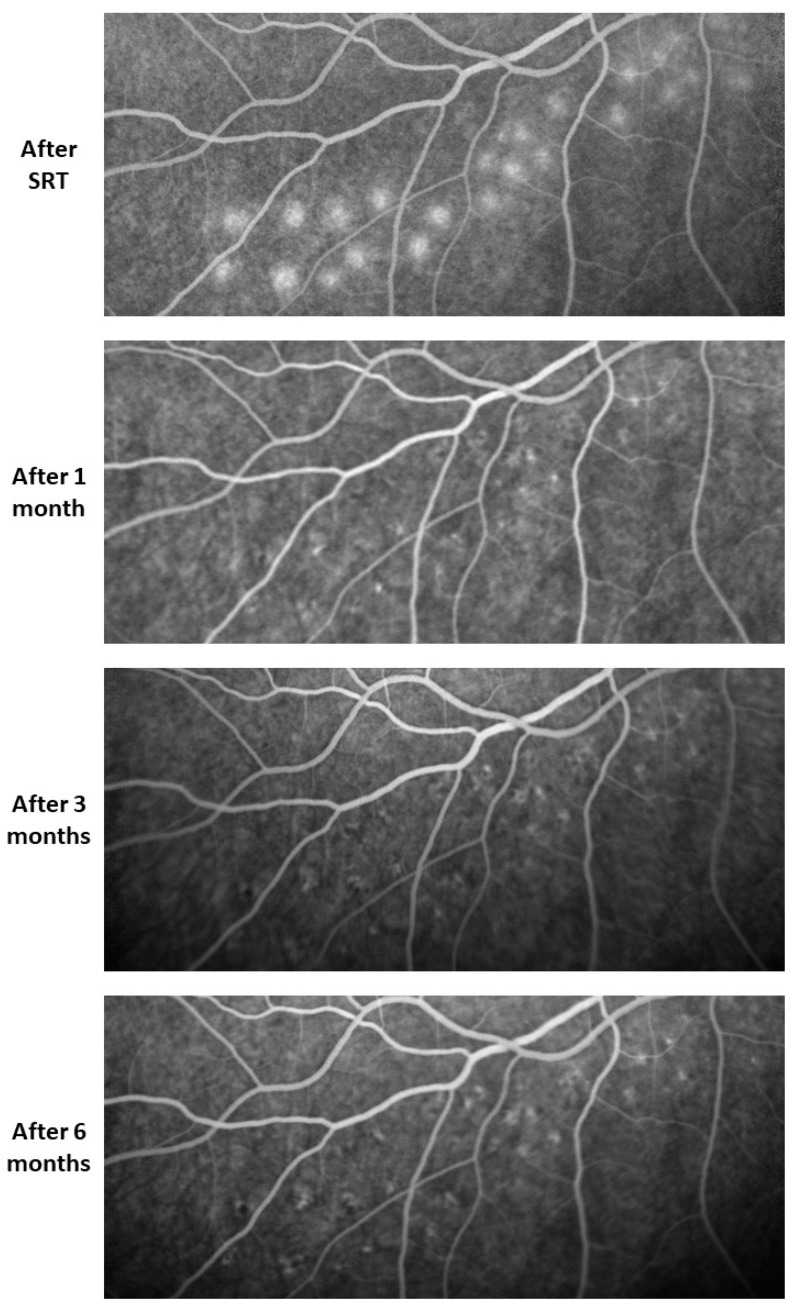
FA images of the titration lesions over 6 months. Leakage that indicated RPE cell death was seen directly after SRT. No more leakage was visible 1 month after treatment. Hyperfluorescent spots interspersed with hypofluorescent areas can be observed within the titration lesion area. It was observed that these lesions shrink over time.

Leakage and thus the destruction of RPE cells were observed in FA performed immediately after SRT at the lesions where higher energy was used. One month later, there was no longer any leakage visible in FA. Hyperfluorescent spots interspersed with hypofluorescent areas were observed. These changes diminished over the months (Figure 5).

**Figure 6:**
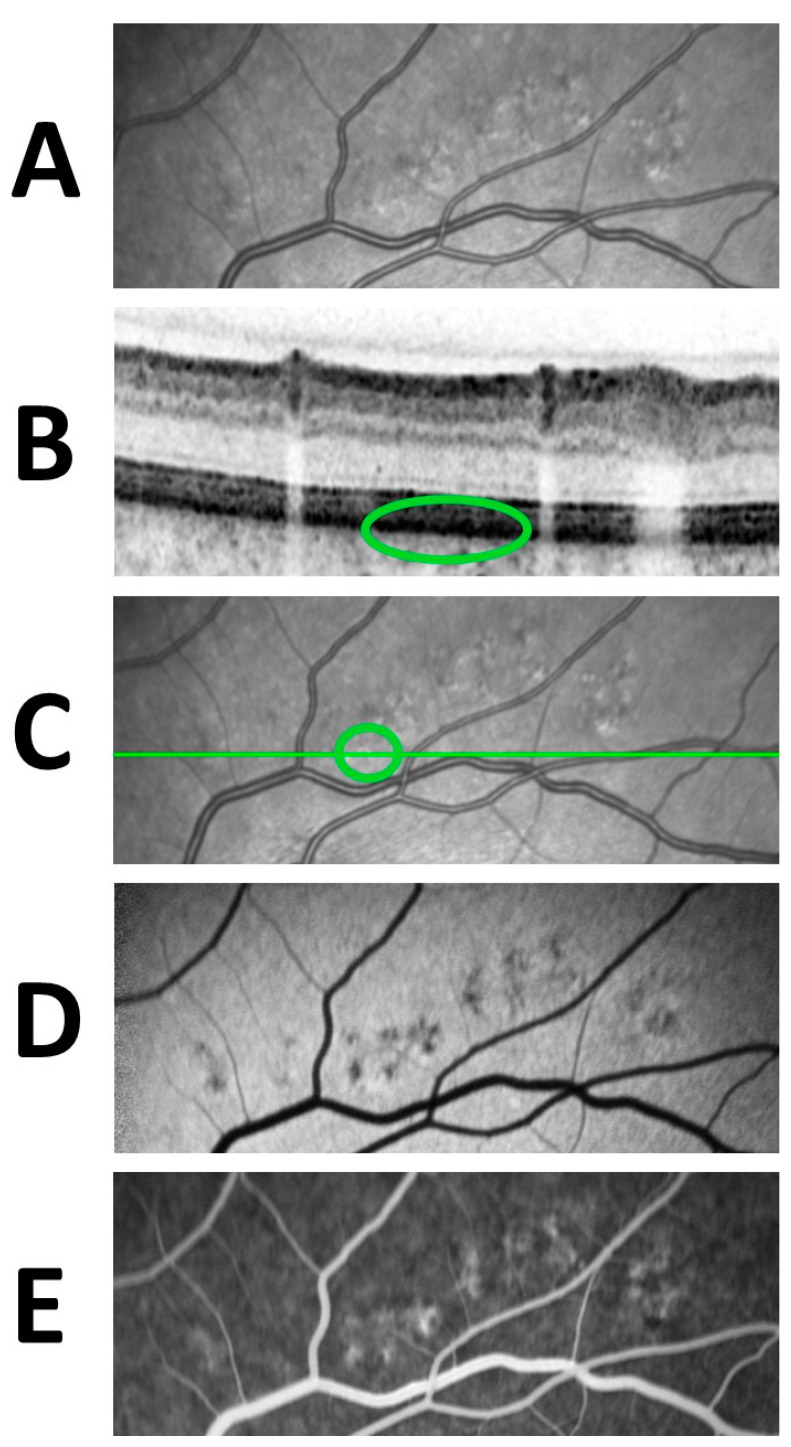
Multimodal imaging of SRT titration lesions 1 year after treatment. The lesions appear as regressive hyperreflective spots in the infrared image (**A**). OCT (**B**) shows RPE hypertrophy with an adjacent RPE defect (green circle) at the site of lesions which are seen as hyperreflective spots in the infrared image (**A**) The location of the OCT section is shown as the green line and at the hyperreflective spot is shown within a green circle (**C**). The lesions appear as hypoautofluorescent areas in FAF (**D**) and hyperfluorescent areas in FA (**E**).

Taking into consideration the combined changes in various imaging techniques, it was observed that the hypo-autofluorescent areas seen in FAF are seen as hyperfluorescent areas in FA (Figure 6). These transmitted choroidal hyperfluorescence, so-called window defects, decreased in size during the follow-up.

### 3.4. SRT Lesion Characteristics

An average of 27.6 ± 7.0 test lesions was applied (minimum 14, maximum 42). Of all the applied lesions, 15.9 ± 8.3 (58 ± 28%) were visible in the initial fluorescein angiography performed directly after irradiation. The fluorescein angiography directly after SRT showed no visible test lesions in three cases. No initial fluorescein angiography was performed on three patients due to earlier described adverse effects following FA.

**Figure 7:**
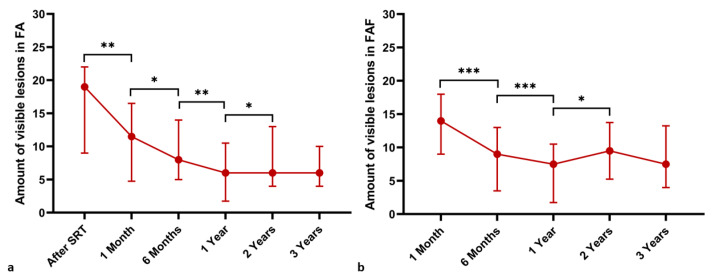
Decrease in the number of visible titration lesions in FA (**a**) and FAF (**b**) over time. Median values are plotted and IQRs are displayed as error bars. A Wilcoxon rank test was used to compare means of not normally distributed matched samples. * *p* < 0.05, ** *p* < 0.01, *** *p* < 0.001.

The number of visible SRT test lesions in FAF and FA was examined at the baseline, at one- and six-month as well as one-, two- and three-year follow-up examinations. Fundus autofluorescence was performed prior to the SRT treatment at the baseline but was not repeated right after the procedure. A significant decrease in the number of visible test lesions in FA and FAF was observed between each follow-up examination for up to two years (*p* < 0.05). Changes in the number of visible lesions in the baseline and follow-up FAF and FA examinations are presented in Figure 7 and Table 1.

**Figure 8:**
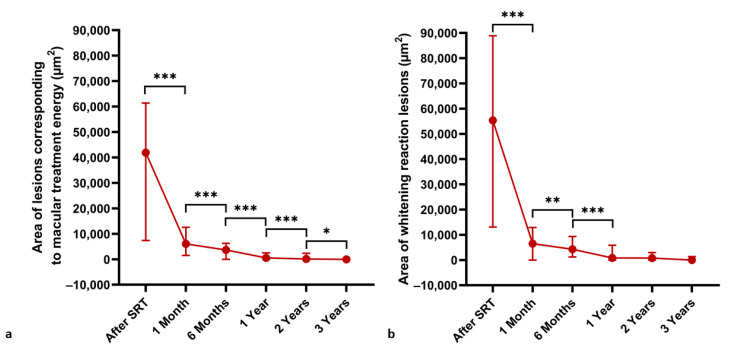
Decrease in area (µm^2^) irradiated with energy levels suitable for macular treatment (**a**) and visible whitening reaction (**b**) over time. Median values are plotted and IQRs are displayed as error bars. A Wilcoxon rank test was used to compare means of not normally distributed matched samples. * *p* < 0.05, ** *p* < 0.01, *** *p* < 0.001.

The SRT spot size was measured over time at titration lesions corresponding to macular treatment energy and lesions with visible whitening reaction for titration purposes (Figure 8). A steady significant decrease in the area (µm^2^) of lesions corresponding to the treatment energy was observed between all examinations (*p* < 0.05). Meanwhile, the area (µm^2^) of visible whitening reaction lesions decreased over time up to the one-year follow-up examination and then stabilized (*p* < 0.01).

Correlation analysis showed no significant correlation between the energy used for macular treatment and the size of the corresponding titration lesions at one-, three-, six-, twelve- and twenty-four-month follow-up examinations (*r* = −0.14–+0.09; *p* > 0.3). Subject age showed no significant correlation with the amount of energy required to see a visible whitening reaction of the titration lesions (*r* = 0.08; *p* = 0.507), treatment energy (*r* = 0.14; *p* = 0.227) or the number of visible lesions in FA at the baseline (*r* = −0.16; *p* = 0.212). Age was not associated with area of lesions corresponding with macular treatment energy at the baseline or any of the follow-up examinations (*r* = −0.32–+0.05; *p* > 0.09). The Mann–Whitney U test showed no significant difference between males and females in the distribution of visible titration lesions in FA (15.9 ± 8.4; median 19, IQR 13 vs. 15.3 ± 6.9; median 17, IQR 14; *p* = 0.64), the area of lesions corresponding to macular treatment energy (44,331 ± 35,497; median 44,071, IQR 58,478 vs. 29,046 ± 11,686 µm; median 29,633, IOR 23,000; *p* = 0.16), and visible whitening lesions (54,022 ± 38,431; median 55,659, IQR 77,432 vs. 40,947 ± 30,933 µm; median 44,873, IQR 55,504, *p* = 0.41) at the baseline as well as the energy needed to reach the visible whitening effect (179.3 ± 29.5; median 180, IQR 40 vs. 177.5 ± 31.5 µJ; median 185, IQR 63; *p* = 1.0).

## 4. Discussion

In this study, the effect of SRT treatment on healthy retina in cCSC was evaluated in multimodal imaging over time. Analysis of OCT images showed dune-shaped thickenings in the RPE layer and irregularities in the interdigitation zone one month after SRT treatment. All of these changes regressed over time and normal morphology of the retina in OCT images was observed in an average of 17.7 ± 10.1 months. Our study shows no evidence of RPE atrophy after SRT irradiation. Correlation analysis showed no correlation between absolute energy and the effect of SRT. This shows that there is no critical absolute energy needed to see the effect of SRT and underlines the importance of titration because different energy values are needed in each individual due to anatomic differences, for example, in the pigmentation of the fundus. Furthermore, age and sex had no influence over the SRT effect; therefore, there is no need to adjust the treatment energy for these variables.

As previously reported in various animal studies, histological examinations have shown that RPE defects caused by performing SRT laser treatment are closed through hypertrophy and migration of the neighboring cells. This remodeling process usually lasts about one week or up to four weeks in the longest cases yet reported [7,9,24]. Thickenings in the RPE layer observed in OCT images of our subjects were consistent with the findings reported in animal studies. Consequently, these data suggest that an analogous remodeling process occurs in both humans and animals. However, morphological changes in OCT images were observed over a significantly longer time period in the subjects of this study. This indicates that the regenerative process of the RPE and retina induced by SRT treatment takes longer in humans than in animals. This is also consistent with another study showing changes in OCT morphology for up to six months in patients treated with SRT [12].

Alterations were never seen in fundus photography. Changes seen in OCT correlated with altered areas observed in FA and FAF, that were also visible in infrared and multicolor images. The FAF technique is based on the emission of short wavelength autofluorescence by lipofuscin in RPE cells. As a consequence of photoreceptor phagocytosis, lipofuscin accumulates in RPE cells [27]. Thus, FAF is an indicator of metabolism in the cells [15]. As previously reported, decreased FAF can be detected immediately after SRT treatment. Framme et al. attributed this effect to a blockade to autofluorescence caused by the destruction of RPE cells and a focal edema [27]. This provides a good explanation for reduced FAF in the areas where SRT was performed, because edematous changes at the tips of the outer photoreceptor segments were also found in histological examinations up to seven days after SRT laser treatment in animals [9,24]. Furthermore, lipofuscin in a solution, after RPE cell destruction, shows decreased autofluorescence [26]. This could also partly explain the initial decline in FAF. In this study, a decreased FAF was also observed after SRT and can be explained by RPE destruction and an intraretinal edema, as mentioned above.

Many studies observed a switch in FAF after one week; lesions showed increased autofluorescence and remained stable for up to 15 months. The authors attributed this to an increased RPE metabolism and the corresponding increase in fluorophore concentration [27]. However, the reported descriptions of FAF changes one month after SRT are mixed. Another group found increased FAF at the areas of irradiation only in a few lesions three months after SRT was performed [11]. Other studies reported a disappearance of the lesions in FAF after three months, but the subtle changes described in our work can still be seen in the figures of these studies [12,14]. None of these studies provided an explanation for the mixed autofluorescence in the treated spots which sometimes persist over several years.

In this study, a complete or partial increase in FAF of some SRT lesions was observed after one month. However, at six months and onwards the same spots showed primarily a decreased autofluorescence with portions of increased FAF. A study on Chinchilla Bastard rabbits revealed a similar freckled pattern of SRT lesions in FAF as early as seven days after SRT [28]. This shows again the analogous processes in both humans and animals, which occur sooner in animals.

Increased FAF may result from an increase in intracellular lipofuscin. This is a byproduct of RPE cell debris being phagocytized by other RPE cells as well as accumulated photoreceptor outer segments being phagocytized by regenerated RPE cells. Microglia originating from the neuroretina and macrophages originating from the choriocapillaris are also attracted and contribute to removal of the debris. This explanation was proposed by previous studies to justify similar changes observed in FAF after conventional laser treatment [12]. Histological studies in animal models showed hypertrophic RPE cells of various sizes and heights with enhanced phagocytic activity, which resulted in intracellularly cellular debris in the first days following SRT irradiation [2,9]. This explains the increased FAF observed after one month in this study. In porcine organ cultures, closure of SRT-induced defects caused by migration and proliferation were observed within five days. However, those cells showed an altered morphology and SRT treatment increased active MMP expression, resulting in the pathologically swollen Bruch’s membrane becoming thinner [6]. It is stated that this leads to a restored and rejuvenated RPE but may also explain altered FAF due to altered morphology and metabolism.

In this study, FA images showed hyperfluorescent areas because of an enhanced transmission of choroidal fluorescence, a so-called window defect, in the areas of reduced FAF. Usually, this corresponds to RPE atrophy [15]. However, in this study OCT imaging showed no RPE atrophy in the irradiated areas. This phenomenon could be explained by findings from cell culture studies. When pigmented primary human RPE cells are cultured, they rapidly lose pigment granules as they are shared between daughter cells; this occurs because critical components of the melanogenesis pathway are no longer expressed in adult human RPE [29]. Animal studies also described a layer of hypopigmented and hyperpigmented RPE cells with apical melanin granules covering the defect 14 days after SRT [2,9].

A combination of the above-mentioned factors could explain the altered FAF and window-defects. Following SRT, the RPE defect is closed by the hypertrophy and migration of surrounding cells. These cells phagocytose RPE debris, resulting in increased lipofuscin uptake, leading to an increase in FAF. However, the regenerating RPE cells show reduced or no intracellular melanin, because adult RPE cells are no longer able to synthesize it. Thus, window defects are in seen in FA in these areas. Furthermore, the rejuvenated RPE cells show an increased active MMP expression and altered morphology, which also results in a change in FAF. It is particularly important to note that no RPE atrophies developed throughout the observed period after laser treatment.

All of this shows that while changes in the FAF are observed over three years, they do not represent RPE atrophy but rather regenerative processes. The RPE monolayer regenerated and photoreceptors remained intact in all cases during the observation period of three years. In contrast to continuous-wave laser treatment, where scarring processes and expanding atrophic creep are observed, changes in the FAF after SRT have even been shown to shrink over time. Interestingly, this also applies to the irradiated spots with a barely visible whitening reaction, which are necessary for titration. This indicates that SRT may have an even wider therapeutic range than previously assumed.

SRT has been shown to thin a pathologically thickened Bruch membrane, regenerate the rejuvenated RPE and lead to decreased vascular endothelial growth factor (VEGF) release and increased release of the VEGF antagonist, pigment epithelium-derived factor (PEDF) [6]. Thus, SRT could also be a promising therapy option for age-related macular degeneration (AMD). However, it is essential to ensure that no damage to the RPE and photoreceptors occurs through treatment when applying irradiation in the fovea. The LEAD trial treated AMD patients using a 532-nm Q-switched neodymium-doped yttrium–aluminum–garnet laser with ultra-short 3-nanosecond pulse durations along the vascular arcades. Not only did the study fail its primary endpoint of inhibiting conversion of intermediate to neovascular AMD, the treatment also led to deep retinal hemorrhages in 6.8% of the participants [30]. This results in photoreceptor and RPE damage and can potentially lead to the development of neovascularization, and therefore is contraindicated for macular and foveal treatment. In contrast, SRT has already been shown to slow the progression of intermediate AMD by improving mean retinal sensitivity measured by microperimetry and reducing the rate of change in drusen load in a pilot study. However, due to safety concerns, treatment was performed at least 1500 μm away from the center of the fovea in this study [7]. Nevertheless, the results of our study emphasize the absence of RPE and photoreceptor damage even three years after SRT and thus support the idea that early maculopathies and other pathologies in the fovea may be treated with SRT as well.

## 5. Conclusions

Photodisruptive SRT is a laser technology selectively targeting the RPE and sparing surrounding tissue such as the neurosensory retina. It has already been used in patients with different macular diseases without inducing scotoma or other side effects due to causing no thermal damage. In contrast to conventional laser coagulation therapy, no atrophies could be detected after SRT, and the induced alteration in RPE cells returned to a normal morphology and metabolism over time. This study demonstrates that a similar remodeling process is observed in humans to the ones depicted in animal studies. However, in humans this process happens over a much longer time period than expected from animal studies. Furthermore, even over a long observation period of three years alteration in FAF can be observed with an absence of corresponding RPE atrophies. The changes in FAF and FA can be explained by altered cell morphology, increased intracellular lipofuscin due to phagocytosis of RPE cell debris, lack of melanin synthesis in adult regenerating RPE cells, and altered metabolism with increased active MMP expression. This absence of RPE and photoreceptor damage even three years after SRT suggests the possibility of a safe therapeutic application of SRT in the macula and fovea. This indicates that there may be further applications of SRT in the future, such as treatment of early stages of AMD and diabetic macular edema to prevent disease progression, in addition to treatment of CCS.

## Figures and Tables

**Table 1:** Number of visible lesions in FA and FAF at the baseline and different follow-up examinations.

Follow-Up Examination						
	Baseline	1 Month	6 Months	12 Months	24 Months	36 Months
Visible lesions in FA						
Number of subjects	33	14	27	26	15	7
Mean ± SD	15.9 ± 8.3	10.6 ± 7.1	9.2 ± 5.5	6.6 ± 5.3	7.8 ± 5.3	6.3 ± 3.7
Median (IQR)	19 (13)	11.5 (12)	8 (9)	6 (9)	6 (9)	6 (6)
Minimum–Maximum	0–29	0–21	0–20	0–18	0–18	0–11
Visible lesions in FAF						
Number of subjects	X	33	33	30	16	10
Mean ± SD	X	12.7 ± 6.4	8.9 ± 5.7	7.1 ± 5.5	9.1 ± 5.4	8.2 ± 5.6
Median (IQR)	X	14 (9)	9 (10)	7.5 (9)	9.5 (9)	7.5 (9)
Minimum–Maximum	X	0–23	0–19	0–18	0–16	0–16

## Data Availability

The data that support the findings of this study are available from the corresponding author, MB, upon reasonable request.
